# Critiquing imaginaries of ‘the public’ in UK dialogue around animal research: Insights from the Mass Observation Project

**DOI:** 10.1016/j.shpsa.2021.12.009

**Published:** 2022-02

**Authors:** Renelle McGlacken, Pru Hobson-West

**Affiliations:** School of Sociology and Social Policy, Law and Social Sciences Building, University Park, University of Nottingham, Nottingham, NG7 2RD, United Kingdom

**Keywords:** Publics, Animal research, Imaginaries, Knowledge, Mass observation project

## Abstract

With an established history of controversy in the UK, the use of animals in science continues to generate significant socio-ethical discussion. Here, the figure of ‘the public’ plays a key role. However, dominant imaginaries of ‘the public’ have significant methodological and ethical problems. Examining these, this paper critiques three ways in which ‘the public’ is currently constructed in relation to animal research; namely as un- or mis-informed; homogenous; and holding fixed and extractable views. In considering an alternative to such imaginaries, we turn to the Mass Observation Project (MOP), a national life-writing project in the UK. In its efforts to generate writing which is typically reflexive, its recognition of the plurality and performativity of identity, and embrace of knowledge as situated yet fluid, the MOP offers lessons for approaching views towards animal research and the role of publics in dialogue around the practice. In considering the MOP, we underline the need to acknowledge the role of method in shaping both what publics are able to articulate, and which positions they are able to articulate from. Finally, we stress the need for future dialogue around animal research to involve publics beyond one-way measurements of ‘public opinion’ and instead work to foster a reciprocity which enables them to act as collaborators in and coproducers of animal research policy, practice, and dialogue.

## 1 Introduction

Across the social sciences, the scientific use of animals has generated a rich body of research. Attracting a diverse range of scholars, animal research has been studied as a scientific controversy (Nelkin, 1995), a space in which human and non-human actors intersect through science (Birke et al., 2007), and most relevant to this paper, a socio-political issue in which public opinion is enrolled as currency (Hobson-West, 2010). Indeed, as Davies et al. (2020, 3) have commented, the plurality of reasons drawing those to the study of animal research reflect its ‘material importance and imaginative pull […] as a space for studying the remaking of human-animal relations and ethical practices in an era of modern biomedical science’. Authors have particularly focused on the practices and relations inside the laboratory and have complicated assumptions of interspecies relationships as straightforwardly exploitative. Indeed, studies (Greenhough and Roe 2011, 2018a, 2018b; Giraud & Hollin, 2016; Friese & Latimer, 2019) reveal the complicated ways in which caring and killing coalesce in the name of science, affecting all those involved in varying ways.

Whilst physically outside the laboratory, the role of publics remains crucial to the ongoing social legitimisation of animal research (Hobson-West & Davies, 2017). This is made explicit in recent emphasis placed on openness in the bioscience sector (UAR 2014). This openness agenda is intended to promote increased interaction with ‘the public’, encouraging research institutions to be more transparent and communicative about their animal use. However, this raises some key questions, particularly around how ‘the public’ is imagined.

The aim of this paper is therefore to provide a critical sociological analysis of the ways in which publics are currently imagined in dialogues around animal research. To achieve this aim, we draw on work from across the field of critical Public Understanding of Science which has thoroughly problematised the framing of public understandings of technoscientific issues as marked by a deficit of knowledge (Millar & Wynne, 1988; Wynne, 1992) or deficit of trust (Hagendijk, 2004; Irwin, 2006; Wynne, 2006). In brief, this work critiques the assumption that once educated or informed, laypeople will accept ‘expert’ opinion and their concerns will be resolved. As this paper will show in relation to animal research, such deficit-model approaches are entrenched in, and made possible by, particular imaginaries (Jasanoff & Kim, 2009; Hilgartner, 2012) of ‘the public’. To support our argument we draw on the example of the UK's Mass Observation Project (MOP) to illustrate that alternative ways of thinking about ‘the public’ are possible.

Based in The Keep, an archive held at the University of Sussex, the MOP is a national life-writing project that, as stated in its tagline, records ‘everyday life in Britain’. The MOP functions by sending out three ‘Directives’ (a set of questions or prompts on a particular topic) per year to its panel of voluntary correspondents known as ‘Mass Observers’ who live across the UK and submit their writing in either paper or electronic formats. In early 2019, there were 310 active writers on the panel, a high representation of whom are located in South East England, are female, and are over the age of 61 (Mass Observation, 2019, pp. 1–4). Topics for Directives are diverse, ranging from the FIFA World Cup to climate change, yet are brought together under the heading of ‘everyday life’. Most Directives are internally designed using input from archival staff, although the MOP also accepts commissions from external researchers and suggestions from Observers themselves (Bloome et al., 1993). MOP writings have been used for social scientific studies across research areas, with academic papers based on analyses of MOP materials covering topics as wide-ranging as gardening practices (Bhatti, 2014), interspecies kinship (Charles, 2014), and genetics and cloning (Haran & O’Riordan, 2018).

As this paper will demonstrate, with its ability to capture thought as a continual process, recognition of identity as performative and relational, and its embrace of the temporality and locatedness of knowledge and views, the MOP challenges many of the assumptions underpinning dominant imaginaries of publics and their views mobilised in the science-society dialogue around animal research. Further, in recognising its panel of correspondents as coproducers of the overall project of Mass Observation rather than simply participants, the MOP offers lessons for how we might conceptualise publics as stakeholders in the societal project of negotiating the scientific use of animals. Therefore, unlike other common qualitative methods such as interviews which often limit participants to the role of respondent, the MOP can provide methodological guidance of specific importance to dialogue around animal research.

In discussing the MOP's potential as an alternative to dominant imaginaries of ‘the public’ and treatment of ‘public’ views and opinion, we present analysis of MOP writing on the 2016 Directive on the topic of ‘Using animals in research’. This analysis draws from a larger project (McGlacken, 2021a) in which the first author analysed the Directive's 159 responses (72 paper and 87 electronic) using a constructionist thematic approach, which, as Braun and Clarke (2006, p. 81) describe, ‘examines the ways in which events, realities, meanings, experiences and so on are the effects of a range of discourses operating within society’. This project was part of a programme of social science research (Davies et al., 2020, pp. 1–13) seeking to explore the complex entanglements between animal research policy and practice.

The paper is divided into three sections which mirror key themes concerning dialogue around animal research. First, we assess the imaginary of publics as uninformed or misinformed by examining the MOP's ability to capture thinking through writing, demonstrating the reflexive process of thought. Second, we consider the imaginary of publics as a homogenous collective and contrast this with performative and relational understandings of identity. Third, we critique assumptions that public views are fixed and extractable with reference to the MOP's embrace of temporality. Overall, this critique implies that to foster meaningful dialogue around animal research, such imaginaries of ‘the public’ must be replaced with those which recognise publics as stakeholders and collaborators in the ongoing societal project of negotiating the use of animals in research. When discussing particular MOP excerpts, we refer to Mass Observers by the identification numbers they are issued by the archive.

## 2 Imagining publics as un-or misinformed about animal research

In 2014, fuelled by suspicions of secrecy in the bioscience sector (Ipsos MORI 2013), the Concordat on Openness on Animal Research was launched by the research advocacy organisation Understanding Animal Research (UAR). The Concordat has now been signed by over 120 life science institutions in the UK, with signatories pledging to ‘be more open about their use of animals in research’ (UAR 2019). More specifically, signatories have agreed to the four commitments of the Concordat: to ‘be clear about when, how and why we use animals in research’, ‘enhance our communications with the media and the public about our research using animals’, ‘be proactive in providing opportunities for the public to find out about research using animals’, and ‘report on progress annually and share our experiences’ (ibid). With the bioscience community's shift from an emphasis on the risks of openness to the risks of secrecy, life science institutions have attempted to remodel themselves to fit within dominant governance discourses and this new emphasis on transparency. This shift moves ‘the burden of public scrutiny from those who are open about their work, to those who choose to remain guarded’ (Davies et al., 2020, p. 8).

However, the enactment of such openness around animal research has itself come under scrutiny, and has indeed been characterised as a ‘selective openness’, by being primarily ‘a matter of controlling information […] but also a matter of who is to provide information’ (Holmberg & Ideland, 2010, p. 365). As an example of this, Pound and Blaug (2016, p. 168) note that during the development of the Concordat, a public consultation identified that openness would be best guaranteed via inspection of animal research practices by those interested *only* in the welfare of laboratory animals. However, this requirement was ultimately left out of the Concordat. Such openness, therefore, appears to be strategic in practice and potentially more limited than many might assume. Moore's (2017, 427) critique of contemporary transparency discourses within governance structures is apposite here, when they argue that such openness discourses tend to ‘conceive of the public through the lens of trust, in terms of a problem to be solved rather than a co-participant in the creation of an open society’. In such framings, the role of publics is restricted to that of distant and passive witness of the information that institutions choose to make available.

For our purposes, it is thus important not just to explore the practicalities around claims to openness, but also the underlying assumptions about the ‘audience’ for such an agenda. As McLeod and Hobson-West (2015, p. 801) have previously argued, different stakeholders have high hopes for what openness initiatives will achieve, with transparency being constructed as ‘a counter to secrecy’ by animal protection groups, a ‘counter to misinformation and misunderstanding’ by the animal research community, and a ‘counter to public mistrust’ by government and research funders.

In the animal research domain, deficit-model approaches to the contributions of publics are widespread. For example, key authors have argued that increasing public awareness of the regulatory framework will foster public support for animal research (Festing & Wilkinson, 2007). Likewise, campaign groups supporting the use of animals in research, such as Understanding Animal Research, claim that ‘much opposition to animal research is based on misinformation’, and it is therefore ‘necessary to be open and informative in our public messaging about how animal research is conducted with ethical oversight and regard for the 3Rs’ (UAR 2019, 2). Such discourses don't just describe the issues at stake; rather, particular (and restricted) imaginaries of the role and character of publics are being performed (Wynne, 2006, p. 212).

Our analysis shows that similar framings of publics are also employed by organisations invested in the replacement of animal models. For instance, in their report on ‘mapping public perception on animal testing’, tellingly entitled ‘Fact or Fiction?’, The Fund for the Replacement of Animals in Medical Experiments (FRAME) aimed to ‘drill down into the detail of public understanding and perceptions – as well as misconceptions – around animal testing and animal use in research’ with key foci including measurement of ‘knowledge of the regulations’ and ‘awareness of alternatives’ (FRAME, 2020a, p. 6). Indeed, discussing the survey's finding, the CEO of FRAME states that ‘even though there have been scientific advances in recent years and some improvements in regulation, there are still many misconceptions about the use of animals in testing and research’ (FRAME, 2020b). Likewise, international animal rights organisation People for the Ethical Treatment of Animals (PeTA) construct an unaware public in their appeal for transparency in the sector: ‘Help us put pressure on the UK government to lift the veil of secrecy […] because granting the public the right to know what horrible experiments are happening behind closed doors is the first step towards stopping the cruelty altogether’ (PeTA, 2018). As made explicit in this example, once provided with opportunities to know more about animal research, the website presents the assumption that such a public will support PeTA's campaign for its abolition.

Alongside the treatment of publics as lacking the ‘proper’ knowledge on animal research, being uninformed or misinformed, the same data on what publics think or feel about the issue is commonly invoked as evidence of *both* public support of and opposition towards animal research. In the UK, an influential bi-annual ‘attitudes to animal research’ opinion poll is commissioned by government departments and carried out by Ipsos MORI. Reporting on the findings of the 2018 poll, PeTA summarised that ‘The British Public Supports Non-Animal Research’ (PeTA, 2019). In relaying responses to different questions in the poll, they describe that a ‘staggering 75 per cent of respondents call for more work on non-animal approaches’, ‘sixty-six per cent are concerned about the use of animals in experiments’, ‘thirty-two per cent don't trust the regulatory system that governs animal experimentation’, and ‘support for an outright ban on animal experimentation is at a 16-year high, at 27 per cent’ (ibid). However, citing the same poll, UAR concluded that ‘public acceptance of animal research remains high but is conditional on research being conducted for scientific and medical purposes and with high animal welfare standards’ (UAR 2019). Having stated this, UAR claimed that the poll results ‘supports our experiences across UAR that much opposition to animal research is based on misinformation’ (ibid). Whilst PeTA and UAR have very different positions in relation to the ethical and scientific acceptability of using animals in research, what this brief example shows (and see Hobson-West, 2010) is that many stakeholders in dialogue around animal research have much in common in relation to their construction of ‘the public’.

Overall, it is clear that the deficit-model is alive and well in this domain. However, our interest in this paper concerns the implications of these underlying assumptions. In short, we concur with Irwin's (2014, 73–74) claim that ‘[t]he characterisation of certain social groups as operating in a deficit of one kind or another is also a way of positioning one's own competence and authority, and of defining what the ‘core’ issues might (and might not) be'. In our case, this point suggests that other potential ways of understanding (or contesting) animal research that stem from value systems other than science are seen as less credible. Apparent public deficits in knowledge, or deficits that arise from lacking the ‘right’ knowledge, therefore work to prioritise the expertise or authority to speak of particular types of actors, in this case, both science organisations and animal protection organisations. Such constructions of publics are extremely significant, as who gets to speak, and hence which values are represented, has considerable implications for how animal research science is governed and managed. To cite Hilgartner, we thus aim to identify how this public imaginary serves to ‘empower and disempower’ various publics' views on animal research in particular ways (Hilgartner, 2012, p. 190).

### 2.1 Mass Observation and public knowledges

But what might an alternative imaginary look like? In seeking better ways of engaging with the voices and views of publics on animal research, we suggest that the Mass Observation Project can offer useful guidance. With its strong commitment to the importance of embodied knowledges, documenting observations, feelings, and experiences which are lived and located in everyday life, the MOP complicates assumptions of public ignorance or misinformation. Indeed, with the MOP generating writing that is often highly reflexive, Mass Observers frequently critically consider the epistemic value and limits of their own knowledges. Indeed, as Kramer (2014, p. 5) summarises, ‘the strength and richness of Mass Observation here is not just that it is able to reflect the perspectives and experiences of a wide range of people, but also that Mass Observers carefully identify the limits of their knowledge’. Such reflexivity challenges deficit-model framings of public contributions, complicating interpretations of public concerns as symptoms of being naively misinformed or simply unaware. Rather, in identifying not only what they know and do not know about a topic but also offering an appraisal of what such knowledge *means* to them, Mass Observers are able to locate their views within their particular, yet shared, social worlds. In this way, MOP writing has the potential to challenge the authority of scientific knowledge by locating it amongst other ways of knowing that may have similar or, at times, more relevance.

To illustrate these points about lived understandings and reflexivity, we now turn to MOP writing on the topic of ‘Using animals in research’. In the following excerpts, Observers can be seen as questioning themselves as they write. For example –

‘I think the more intelligent the animal is, the worse it is to experiment on them. This is probably illogical and anthropomorphic – how do I know whether a monkey is cleverer than a rat?’ (Mass Observer C5847).

Similarly, another Observer (Z2276) reflects on their feelings towards medical research using animals, finding their hopes for the practice to be ‘a bit of middle class kidding myself’, a form of wishful thinking which they suggest is unlikely to match the reality –

‘I feel grateful that the research has been done and simultaneously slightly guilty about it. Hope it was done in as respectful way as possible, but that's probably a bit of middle class kidding myself and a luxury I shouldn't wallow in. If I'm honest with myself I have to acknowledge that some animals were probably in fear and pain to enable the medicines to be developed.’ (Mass Observer Z2276).

Such brief examples show that, in writing, Mass Observers often record their views and feelings whilst simultaneously assessing their merit demonstrating thinking as a process rather than thought as a final and complete outcome Hobson-West et al., 2019, McGlacken, 2021. In allowing correspondents space to reflect on their own views, the reasons for them, and whether they hold up to even their own personal scrutiny, approaches which frame their contributions to animal research dialogues as marked by a deficit of knowledge look even more hollow. Rather than signalling a lack of information to make a choice one way or the other, the uncertainty and inconsistency of opinion expressed in MOP writing, which Mass Observers themselves often reflect on, are integral to making sense of complex moral issues such as animal research. In this way, the MOP offers the flexibility for individuals to provide their own assessments of what they know, think, and feel about a topic, as well as what they do not. Crucially, this avoids reducing ‘understanding’ to a matter of ‘facts’. As Bucchi and Neresini (2008, p. 451) argue, ‘[f]actual information is only one ingredient of lay knowledge, in which it interweaves with other elements […] to form a corpus no less sophisticated than specialist expertise’.

Furthermore, once we move away from the focus on providing ‘facts’, as a way for stakeholders to ‘enlist’ publics to a particular ‘side’, we can start to ask more complex questions about the extent to which publics themselves consider the question of knowledge, and, for example, how knowledge on animal research may be felt as uncomfortable and actively negotiated (McGlacken, 2021b). Here we agree with Stilgoe et al. (2014, 7) who argue that when thinking about public engagement we first ‘need to know more about fatalism with respect to science governance and disenchantment about engagement, and question the constructed publics that are being invoked in the discourse and practice of engagement’.

## 3 Imagining publics as constituting a homogenous collective

Our second observation is that current policy and stakeholder discourses on animal research imagine the ‘general public’ as a pre-existing, homogenous collective, separate, and separable from other stakeholders. This is exemplified in in UAR's 2019 annual Concordat report, in which they appear to imagine a singular public, united in its physical and professional exclusion from science. For example, ‘this section of the Concordat changes each year as new initiatives are developed and researchers become more assured that the public they will be speaking to is not hostile’ (Williams & Hobson, 2019, p. 31). This construction of ‘the public’ then allows for authoritative claims to be made about what ‘the public’ knows, thinks, feels, or wants. For instance, in their summary of a meeting at which representatives of Concordat signatories discussed challenges of communicating harms, UAR advise that ‘when asked, the public say that they do not necessarily want the gory details of animal research, but would like more information about the harms involved. When they are provided with information they inevitably tell us it is not as bad as they thought’ (UAR 2018, 3).

As with our first imaginary on knowledge deficits, this representation of publics as a homogenous public body is not only articulated by those in the bioscience community. Online animal advocacy group materials often suggest a public imagined as uniform. For example, in 2015, Cruelty Free International's (CFI) Director of Policy claimed that ‘[e]very year millions of animals are used in experiments in the UK, but *the public* knows very little about what happens to animals in research laboratories - or why’ (Cruelty Free International, 2015; emphasis added).

Boundaries are also drawn between ‘the general public’ and categories such as campaigners. For example, in recruiting participants for ‘public dialogue’ workshops intended to feed into the Concordat, Ipsos MORI state that they ‘screened out those who were actively involved in animal rights or animal research, or particular experts on the topic at hand’ (Ipsos MORI 2013, 12). This screening out was noted by staff in the RSPCA Science Group (Jennings & Hawkins, 2015, p. 2) as important in order to create a ‘useful sample of a genuine 'general public'’. Indeed, as Ipsos MORI themselves put it, this screening worked to ‘gather a heterogeneous group of the public’ which ‘did not include *unusually informed* people’ (ibid, emphasis added). Such distinctions are also visible in documents such as UARs ‘Researcher's Guide to Communications' (2009), which distinguishes between ‘the public’ and ‘antivivisectionists’. (UAR 2009, 4–5). Here, ‘antivivisectionists’ are separated from the wider public whose support is something to be won over from this perceived hostile fringe.

These brief extracts are evidence of clear boundary drawing between those with an excess of relevant knowledge or a particular scientific or ethical positionality, with others who are presumably ignorant, neutral, and disinterested. This exclusion of certain groups from the construction of the ‘general public’ has been identified in other science-society interactions, such as Lezaun and Soneryd's (2007, 294) observation of the ‘peculiar return to the figure of the idiot, the person with no known opinions or unprompted interest in public matters’ in public debate around food biotechnology. Such an imaginary is thus rooted in an assumed ‘professional and political neutrality’ (Davies et al., 2020, p. 7).

However, to assume that publics do not have a pre-existing interest in animal research is to ignore the way in which humans are bound up in multiple ways with the issue. For example, as Gorman and Davies (2019, p. 25) have documented, involving patient groups in shaping research has recently become a key focus for research funders and biomedical institutions. Such efforts reflect that embodied knowledge and ‘lived experience’ is of value for informing the ethical and scientific practice of biomedical research. Indeed, one could argue that UAR's (2016) assumption that improved public awareness will improve overall support for animal research only makes sense because of the material connections that publics (as patients and medical consumers) already have with the practice. Hence, in using polls which reproduce the construction of a public consisting of those with no particular stake in animal research, dialogues in this arena thus ignore the many ways in which publics are already affecting and affected by the practice.

Furthermore, to ‘screen out’ certain people with links to campaign groups from the category of publics represents a political move that echoes Wynne's (2007, 107) distinction between invited and uninvited publics, a framing of participation which ‘implicitly imposes normative commitments—an implicit politics—as to what is salient and what is not’. Building on this work, de Saille (2015, 103) discusses the way in which screening out of some participants as ‘biased’ helps suppress protest (de Saille, 2015, p. 103). However, de Saille goes further to argue that uninvited publics may become ‘unruly publics’, whose ‘insistence on engaging with science on its own terms is vehemently discouraged by policy-makers’ (ibid, 106). In the animal research domain, animal rights organisations fit well into this uninvited category, given the way in which they have historically been characterised as irrational or extremist (Michael & Birke, 1994a; Michael and Birke 1994b; Mills, 2013; Munro, 2005; Yates, 2011).

In summary, what we can observe in the animal research arena, is the elevation of invited publics, or what Michael (2009) has termed ‘Publics-in-General’, and the exclusion of uninvited or unruly publics, or ‘Publics-in-Particular’. However, there are potentially significant long-term costs to this strategy. In excluding certain groups identified as having an explicit stake or position, this might exclude those with ‘precisely those attributes which would enable civil society actors to make meaningful contributions—namely independent knowledge, articulated interests, argumentative skills and political or professional involvement’ (Wehling, 2012, p. 47). To put it another way, campaign groups might be seen instead as a form of ‘counter-publics’ (Warner, 2002) or mobilised publics, who are ‘not only interwoven and continuous with what are often called ‘silent majority’ neutral publics, but, […] are articulating the normative public concerns which are often shared silently, well beyond their own network populations themselves' (Welsh & Wynne, 2013, p. 542). For example, in articulating their position on the treatment of animals, such groups may not only be voicing their own particular values but those identified as in the ‘public interest’ (Raman et al., 2018).

Overall, this section has shown that in the UK dialogue around animal research, the imaginary of a homogenised public body, which can be said to collectively ‘think’, ‘say’, ‘want’, or ‘know’ is common yet highly problematic, as is the way the ‘general public’ is distinguished from campaigning organisations. This construction serves to exclude not just individuals or groups, but also *ideas,* working to privilege particular forms of knowledge. Moreover, in reproducing this unified public entity and the notion of ‘public opinion’ it reifies, stakeholders can make claims to consult ‘the public’, hoping to bolster their agendas with democratic weight. Yet, such gesturing to ‘the public’ to fulfil the requirements of the ‘social contract’ said to guide scientific animal use (Davies et al., 2016) constructs this relationship itself as largely symbolic. Indeed, as Hagendijk (2004, 54) has pointed out, ‘the very diversity of forms, parties, and interest, at play in contemporary science and in the surrounding world, makes the idea of such a contract an insoluble thought-experiment’. Hence, somewhat paradoxically, appeals to an imagined ‘general public’ might actually work to undo appeals to democracy, promoting instead only shallow gestures to an illusory relationship between animal research and wider publics.

### 3.1 Mass Observation and public identity

In its resistance to representativeness and its embrace of particularity and complexity, the MOP can thus offer a useful contrast to the construction of ‘the public’ as homogenous. The panel of Mass Observers is not, and does not claim to be, representative of the UK population (Mass Observation, 2019, pp. 1–4). Crucially, however, the Project is committed to embracing the ‘particularity of respondents’ quotes' (Highmore 2009 quoted by Pollen, 2014, p. 4), given the aim of situating topics in the ‘everyday’ worlds that give them meaning. The particularity of MOP writing thus resists attempts to dissect ‘public opinion’ in accordance with ‘variables’ and ‘influencers’ that can be neatly correlated, as demonstrated in opinion polls and attitudinal surveys.

In addition, Mass Observers are encouraged to reflect on what they see around them, resulting in a complex, dual identity, ‘recounting their personal experiences’ whilst also working to ‘document or 'bear witness' to contemporary social life’ (Kramer, 2014, p. 7). In their consideration of the knowledges and experiences of others alongside their own, MOP writing is thus better equipped to capture the plurality of narrative and knowledge, rather than privileging a singular, unified telling.

Due to the openness of the response format, Mass Observers are also able to articulate the intersections of their identities, foregrounding certain aspects of themselves when they feel it is relevant to do so. In relating to different topics, correspondents continuously reframe and remake their self-identities according to the topic at hand, writing as family members, citizen, patients, scientists, consumers, employees, and so on. Although traditional demographic information (i.e. gender, age, location, etc.) is recorded by the archive, Mass Observers are able to (re)articulate their identities through their writing. Hence, the identities of Mass Observers are best viewed as pluralistic and fluid, being shaped by and emerging through each Directive, which not only guide *what* Observers write about and *who* they write as, but also, as Sheridan (1996) notes, *how* they write it. This reminds us that, ‘[p]eople are not simply the sum of the categories into which they fall’ (Kramer 2009 quoted by Pollen, 2014, p. 4).

That the identities of Mass Observers emerge through writing is visible in the following extracts from responses to the ‘Using animals in research’ Directive. The first Observer positions themselves as vegetarian and the second as a former member of the British Union for the Abolition of Vivisection (BUAV) yet also, as they suggest, conflictingly, a meat-eater. In framing their views on the matter through such positions, Mass Observers highlight how particular identities become relevant in relation to particular issues –

‘As a vegetarian I am saying we should only use animals, any animals, where it is only absolutely unavoidable and unquestioningly beneficial to mankind, and that they are treated in the most humane manner.’ (Mass Observer D4736)

∼

‘I was a member of BUAV in my teens and remain entirely convinced of the argument that whilst some knowledge can be gained, essentially animal research tells you about that specific animal species and that for human medical research development we have to find ways to undertake ethical human research. However, I am a meat eater – so how do I square that with an abhorrence of animal exploitation for research purposes? Frankly I don't – and I struggle increasingly with this contradiction.’ (Mass Observer G4566)

These brief examples help demonstrate that different perspectives are possible, for example by breaking down conceptual barriers between publics and social movements, or by using methods such as the MOP which embrace identity as performative and fluid rather than fixed or stable, and do not employ a unified and homogeneous idea of ‘the public’. Indeed, as analysis of MOP writing on animal research has shown (McGlacken, 2021a), at times, Mass Observers themselves may make distinctions between themselves and the ‘general public’, discussing the latter as a collective they may stand outside of and can critically assess. Furthermore, as we shall see in the next section, the MOP also challenges the idea of ‘opinion’ as a free-floating and fixed phenomenon.

## 4 Imagining publics as holding fixed and extractable views

As noted at the start, one of the most widely referenced datasets for ‘public opinion’ on animal research in the UK is the Ipsos MORI biennial national poll on public attitudes to animal research. The release of these statistics provides a key opportunity for media coverage on the topic (Davies, 2019) and for claims by stakeholders such as UAR about levels of public acceptance (UAR 2014, 5). Without rehearsing long-standing debates about the relative merits of quantitative versus qualitative methods, this section provides a critique of the way in which polls, and claims built upon them, perform a particular imaginary of ‘public opinion’.

One problem with poll data in this domain is the way in which responses are guided into set binaries (acceptance/opposition, for/against) and summarised as what ‘the public’ as a whole *think* or *feel.* For example, Ipsos MORI (2018, 6) report that ‘most of the public accept the use of animals in scientific research for medical and scientific purposes’. However, they also account for the fact that 31% of respondents neither agreed nor disagreed with a statement about trust in regulation as evidence that some respondents ‘do not feel they know enough to give an opinion either way’ (ibid, 29).

What this interpretation fails to acknowledge is that some may feel ambivalent, require more details, be unable or unwilling to express a simple for/against opinion, or that others still might be unable to summarise their views in the ‘fixed response options’ they are given (Ormandy & Schuppli, 2014, p. 401). Indeed, in the case of animal research, the questions asked often constrain responses to issues of animal welfare, regulation, and awareness, preventing the expression of other concerns such as the scientific validity of animal models or the value of certain forms of biomedical research. In contrast to the tendency of macro-level polls to simplify areas of uncertainty, in a Danish study, Lund et al. (2012) found that most participants considered the acceptability of each experiment involving animals individually. This suggests that the contingency and contextuality inherent in socio-ethical thought is lost in polling, which seeks to constrain public understandings of animal research into a for or against binary which is absolute and based in fundamental moral positions.

A second and deeper critique comes from understanding polls themselves as performative. For example, Kramer (2007, p. 7; emphasis in original) has explored the role played by public opinion polls in political debate ‘not simply by representing true/factual ‘public opinion’ but by *creating such a category as public opinion* in the first place’. Warner (2002, 54) makes similar observations, describing how the apparatus of polling creates the public as a ‘social fact’ whilst simultaneously obscuring its constitutive role in calling this ‘public’ into being (Warner, 2002, p. 54). In other words, polls are falsely assumed to be neutral tools, through which views of a pre-existing category of individuals are simply uncovered. This in turn serves to strengthen the myth of the ‘general public’, who are defined by their role as ‘providers of views and attitudes’ (Braun & Schultz, 2010, p. 415). To return to Hilgartner (2012), this last point neatly reveals the way in which imaginaries contain assumptions about what democracy is or should be. In short, if the function of publics is simply to provide ‘views’, this is much more narrow role than envisaged by more deliberate democratic approaches, which do not seek to offer simple resolutions, but enable ‘deliberation itself to deal with the conflicts as they arise’ (Gutmann & Thompson, 2002, p. 172).

Overall, recognising polls as performative helps reveal the way in which polls are used to demonstrate the legitimacy or rationality of particular stakeholder positions (Hobson-West, 2010), or are ways of creating apparent public consensus. Following Lezaun and Soneryd (2007, p. 280), we thus contend that opinion polling on animal research currently functions as a ‘technology of elicitation’, which, along with ‘the cohorts of experts that control their application and interpret their results’ compose ‘a veritable extractive industry’, in which public opinion is something to be produced and won in order to serve pre-defined institutional aims.

To give a more concrete example, public opinion is enlisted to justify particular scientific practices. Whilst choice of experimental organism is complex, it is partly influenced by assumptions about public perceptions of particular species (Dietrich et al., 2019; Message & Greenhough, 2019). Hobson-West and Davies (2017) term this ‘societal sentience’ and show how this operates as a powerful imaginary which influences regulation and daily laboratory practice. Therefore, imagining publics as holding fixed, extractable views (for example on what species deserve more or less regulatory protection) has real and profound consequences for the lives of non-humans in research.

In summary, national opinion polling occupies a key role in the UK societal dialogue around animal research and is cited by stakeholders for a variety of ends. This technology constructs ‘the public’ in a particular way, namely as a singular, homogenous entity with fixed and extractable attitudes. Expressions of ambivalence or uncertainty are less valued and arguably misinterpreted in the reporting of results. More substantially, we can also see polls themselves as performing and creating the ‘public’ entity. Rather than fostering reciprocal dialogical processes in which learning is the aim of *all* those involved, polls and surveys ultimately serve to produce ‘public opinion’ as a resource for stakeholders to make democratic gestures, without having to fully grapple with the complexity of interactions with, and understandings of, animal research.

### 4.1 Mass Observation and public ‘views’

With its embrace of contextuality and recognition of the relationship between personal experience and political forces, the MOP can offer an alternative to such treatment of views and opinion. Coproducing ‘one of the major repositories of longitudinal qualitative social data in the UK’ (Mass Observation, 2015), Mass Observers are effectively engaged in a lengthy conversation, with each Directive response comprising one part of a continuing relationship between the Observers, the archivists and the archive. This creates both opportunities and challenges for those interested in using the MOP as a research method or data source, who must acknowledge and contend with the partiality and fluidity of views expressed in a single Directive response.

Given that MOP responses are generally without a fixed deadline, with Observers encouraged to respond within three to four months (Mass Observation, 2015), the MOP affords its correspondents time and space for deliberating over and articulating their views towards complex topics such as animal research. Indeed, Mass Observers are often reflexive about the shifting nature of their beliefs, values, and feelings and the temporality of their views, for example in the influence of particular life events (Kramer, 2014, p. 7). With the importance of temporality to the MOP, the 2016 ‘Using animals in research’ Directive included a specific question about whether correspondents' views had changed over time. Responses are rich in conveying this temporality and reflection –

‘My own views I think have changed over time; I think I'm more moderate than I used to be. When I was a student, I was concerned about the animal experimentation that went on at the University I attended and I'm pretty sure I felt that it was just wrong, and shouldn't happen. As I suppose often happens, as I've gotten older I can see that things are more complex and, having had relatives benefit from medicines and changes in practices that have happened because of animal experimentation, I can see there are obviously benefits.’ (Mass Observer W5881)

∼

‘I used to be dismissive of issues like this when I was younger but the uncomfortable truth behind such matters becomes obvious with even just a little contemplation. We live in an age of political correctness where the inappropriate use of language against another person is perceived as an outrageous affront and then we absentmindedly dine on animals that have been barbarically slaughtered. If people really are so sensitive and compassionate then how can we still condone the brutal and clinical subjugation of other species, especially when we take such lengths to prevent even the slightest offense to our own species?’ (Mass Observer N5744)

As both of these extracts show, views towards animal research are not experienced as fixed and absolute but are open to transformation throughout one's life. For the Mass Observers above, this can mean growing older and coming to view the issue as complex, identifying and experiencing tangible clinical benefits of biomedical animal use or, on the other hand, finding hypocrisies in the differences between the political and ethical treatment of humans and non-human animals. What both examples here demonstrate is that, in giving time and space for individuals to situate their views on animal research in a particular moment, some methodologies can better attend to the particular experiences and contexts which give animal research meaning in the everyday.

To give another example, discussing their analysis of responses to a 1987 MOP Directive on the topic of AIDS, Cook argues that rather than soliciting direct answers, Mass Observation seeks ‘discursive responses guided by general themes and loose questions. These responses allow us to see something of the complex texture of thought, opinion, and feeling’ (Cook, 2017, p. 248). Paying attention to this ‘complex texture’ is clearly a far cry from the binary positions implicit in the Ipsos MORI animal research polls. It also diverges from using publics to establish evidence or trust for a pre-existing approach (Stilgoe et al., 2014). Rather, given that the Mass Observer role is often characterised as documenting a ‘people's’ history (Bloome et al., 1993), capturing the ‘everyday’ and the ‘ordinary’, recording observations on a given topic can even be an opportunity for Observers to perform their own model of citizenship (Hobson-West et al., 2019). In other words, the role of Mass Observers is not simply to ‘respond’ to a topic, but rather to act as co-producers of the collective project of Mass Observation (Pollen, 2014), informing what should be written about and how their writings should be used (Sheridan, 1993).

## 5 Conclusion

The societal dialogue around animal research in the UK is characterised by a great deal of political, stakeholder, and media attention devoted to calculations of, or reactions to, what ‘the public’ think. We contend that this focus is both methodologically and ethically problematic without a more thorough and critical analysis of who various publics are and how we should attend to their perspectives. Using ideas from critical Public Understanding of Science literature, our theoretical starting point is that particular imaginaries of ‘the public’ feed into the assumptions and claims about what publics think and want, and thus have significant roles to play in influencing the practice of animal research. These imaginaries are interrelated but, for clarity, we have divided these into three dimensions.

First, in demonstrating the prevalence of deficit-model framings of publics and their contributions to animal research dialogues, we aim to contribute to the troubling of the current openness agenda, which tends to assume that increasing public awareness will either assuage socio-ethical concerns or ignite more scrutiny of animal research. As well as being cast as un- or mis-informed, we then showed how publics are often imagined as a pre-existing, homogenous group with a singular identity which is ontologically separable from other groups such as campaigning organisations. This is a powerful imaginary, as it helps to articulate who gets to count as a member of the public, preventing those deemed as *too* interested, involved, or knowledgeable from participating. Finally, we developed a critique of underlying assumptions that public ‘views’ or ‘opinion’ are fixed and extractable via methods such as opinion polling, suggesting instead that such methods work to constitute and reify the notion of ‘public opinion’ and can thus be understood as performative in nature.

Throughout, we have discussed the UK's Mass Observation Project as a potentially useful alternative to these problematic imaginaries of publics and their contributions to societal dialogues. Whilst more detailed analysis of this written material is provided elsewhere, our aim here in including excerpts of MOP writing was to elucidate wider conceptual lessons for those interested in publics and animal research. In challenging the three key issues that structure this paper, we first showed how the MOP encourages reflexive writings and embraces thoughts, feelings, and understandings as emerging through lived social worlds. Second, in resisting the pull of representativeness, the MOP does not try to construct its panel of correspondents as a homogenous collective, and instead recognises the performativity and plurality inherent to knowledge claims and identity formation. Thus the MOP does not seek to reify a false notion of the ‘ordinary’ member of the general public based in knowledge or expertise deficits or political and ethical neutrality. Thirdly, given its longitudinal nature, the MOP attunes us to the temporality and fluidity of views and feelings on an issue, with writing on a given topic being borne from a particular moment and open to rearticulation in the future.

Overall, then, careful use of the MOP can attune us to the performative essence of identity and the relationality of sense-making, and reminds us of the importance of methodology in influencing who gets to respond and how. This analysis demands a radical shift in how research on animal research and publics is conducted and understood, a shift which may unsettle the aim of seeking to understand ‘public opinion’ in the first place.

Without this shift, current science-society relations are falling short of the Concordat's own aim to ‘build open *dialogue* with the public on the reality of the use of animals in research’ (Williams & Hobson, 2019, p. 8, emphasis added). Dialogue requires a reciprocal approach of shared learning, in which scientific knowledge is not privileged above other ways of understandings the issue, including questions of ethics. Such dialogue would also require a shift from imagining publics primarily as respondents and recipients to collaborators in and coproducers of animal research policy, practice, and dialogue.

Discussing openness around animal research, Carbone (2021, p. 16) argues that ‘If the goal is to truly meet a moral obligation of accountability […] science insiders will invite outsiders, including animal advocates critical of the enterprise, to join in the editing process, ask questions, determine for themselves what information they want more of, and beyond that, to collaborate in being part of the story themselves’. As we have suggested throughout, this process of collaboration must also open itself further to the involvement of publics. Moving beyond the imaginaries we have laid out here presents an initial step towards this, encouraging us to talk *with* publics rather than only about them.

## Declaration of competing interest

The Authors declare that there is no conflict of interest.
